# Advances in Cardiac Development and Regeneration Using Zebrafish as a Model System for High-Throughput Research

**DOI:** 10.3390/jdb9040040

**Published:** 2021-09-25

**Authors:** Nicholas Francoeur, Rwik Sen

**Affiliations:** Active Motif, Incorporated, 1914 Palomar Oaks Way, Suite 150, Carlsbad, CA 92008, USA; nfrancoeur@activemotif.com

**Keywords:** cardiac development, regeneration, epigenetics, transgenic reporter, transcriptome, epigenetic, ChIP-seq, ATAC-seq, CRISPR, high-throughput

## Abstract

Heart disease is the leading cause of death in the United States and worldwide. Understanding the molecular mechanisms of cardiac development and regeneration will improve diagnostic and therapeutic interventions against heart disease. In this direction, zebrafish is an excellent model because several processes of zebrafish heart development are largely conserved in humans, and zebrafish has several advantages as a model organism. Zebrafish transcriptomic profiles undergo alterations during different stages of cardiac development and regeneration which are revealed by RNA-sequencing. ChIP-sequencing has detected genome-wide occupancy of histone post-translational modifications that epigenetically regulate gene expression and identified a locus with enhancer-like characteristics. ATAC-sequencing has identified active enhancers in cardiac progenitor cells during early developmental stages which overlap with occupancy of histone modifications of active transcription as determined by ChIP-sequencing. CRISPR-mediated editing of the zebrafish genome shows how chromatin modifiers and DNA-binding proteins regulate heart development, in association with crucial signaling pathways. Hence, more studies in this direction are essential to improve human health because they answer fundamental questions on cardiac development and regeneration, their differences, and why zebrafish hearts regenerate upon injury, unlike humans. This review focuses on some of the latest studies using state-of-the-art technology enabled by the elegant yet simple zebrafish.

## 1. Introduction

Zebrafish is an excellent model system for research on developmental biology, and this model has been used in highly impactful studies that have defined the field. The earliest record of zebrafish development research dates back to 1948, when Battle and Hiasoka investigated the effect of a carcinogen on zebrafish development to understand its impact on differentiation and organization during embryogenesis [1]. In one of the earliest studies on developmental genetics using zebrafish, Streisinger et al. presented the production of homozygous diploid zebrafish clones, which laid the foundation for generating clonal lines and genetic manipulations in developmental research [2]. The study focused on treatments of zebrafish eggs by late and early pressure as well as heat shock, and on treatment of zebrafish sperms by irradiation. In 1995, a study by Kimmel et al. [3] focused on developmental staging and cell lineage mapping in zebrafish, providing the first detailed documentation of the zebrafish development from zygote period at 0 hours to protruding mouth at 72 hours post fertilization (hpf) [3]. Several other seminal studies focused on genetics and mutations in zebrafish [4,5,6] with a focus on the development of specific organs such as the brain [7], heart [8], digestive tract [9], et cetera.

Over the years, there has been a widespread increase in the use of zebrafish as a popular model for research on developmental biology. This popularity can largely be attributed to the presence of human gene orthologs in zebrafish, as well as an abundance of samples due to their large brood sizes and more accelerated life cycle than that of mammals [10,11]. Zebrafish are also amenable to sophisticated microscopy and imaging procedures. Because zebrafish undergo external fertilization and are transparent during early developmental stages, zebrafish growth during specific developmental windows can be monitored in real-time. Zebrafish are likewise advantageous for forward and reverse genetics studies [12], because it is convenient to employ sophisticated techniques to create mutations [13] and transgenic reporter lines [10,14,15]. Elegant mutational techniques such as CRISPR are also easy to employ.

In addition to developmental research, the zebrafish model is extremely popular in the field of regeneration, as the zebrafish can regrow almost any part of its body. Several studies have focused on the regenerative potential of zebrafish to address similar questions in humans, where regeneration is not as widespread. Regeneration in the context of zebrafish, or teleosts which are fish with fully movable maxilla and premaxilla, was first documented 235 years ago [16]. Over the last several decades, many studies have proven zebrafish to be an excellent model for studying regeneration, especially in relation to cardiac biology [10,17,18,19,20,21,22,23].

Overall, zebrafish provide an excellent model for studying phenotypes and molecular mechanisms associated with development and disease, pharmacological treatments pertaining to drug discovery and environmental research, etc., and genetic manipulations [24,25,26]. With increasing focus on genome-wide profiling of transcriptomic and epigenomic landscapes, studies on heart development and regeneration in zebrafish have started employing high-throughput techniques using next-generation sequencing to address the above questions. The zebrafish model has proven to be highly compatible with the new technology, as the large quantity of samples required can be obtained from zebrafish in a very short span of time.

As heart diseases are the primary cause of human mortality in the US according to the CDC (Center for Disease Control and Prevention), future studies of cardiac development and regeneration in any species with associated orthologs will be invaluable to public health. With continuing development and refinement of high-throughput techniques for transcriptomic and epigenomic profiling, animal models compatible with these efforts will be critical in the advancement of basic research. In this review, we explore the specific contributions of the zebrafish model to cardiac development and regeneration research involving state-of-the-art technology for high-throughput transcriptomic and epigenomic profiling.

## 2. Zebrafish Heart Development

For a better context of recent studies using a high-throughput methodology on zebrafish cardiac development and regeneration, an overview of zebrafish heart development is provided, but in brief, because several impactful studies and reviews have already described the process in detail [27,28,29,30,31,32]. Development of the heart occurs earliest among all other functional organs in zebrafish when the cardiac crescent begins to form with contributions from cardiogenic progenitor cells post-gastrulation [33]. Subsequently, the cardiogenic mesoderm gives rise to the first heart field (FHF) and second heart field (SHF) [33,34,35]. The FHF contributes to the primary heart tube, left ventricle, and most of the atria, while SHF contributes to the right ventricle, outflow tract, and atria [33,36,37]. Orchestrated signals from mesoderm-derived FHF and SHF, as well as endoderm, are crucial for cardiogenesis [33]. Broadly, zebrafish heart development comprises of specification and differentiation of myocardial progenitor cells, migration and fusion of bilateral heart fields, and development of endocardium, myocardial tube, cardiac looping, valves, conduction system, and epicardium [23]. Studies on gene regulatory networks for the development of the heart in zebrafish show a complex involvement of multiple genes in the process [6,38,39,40].

Zebrafish have a 2-chambered heart with a single circulation system, whereas humans have a 4-chambered heart and double circulation system. Despite lacking in those complexities, the zebrafish heart is capable of regeneration upon injury, unlike human hearts. In zebrafish, blood flows into a single atrium, which is connected to a single ventricle by an atrio-ventricular valve [41]. The ventricle pumps blood into circulation through a prominent outflow tract called the bulbus arteriosus [10].

The absence of cardiac regeneration in humans has focused the attention of many researchers towards non-mammalian species [10,42,43]. Several studies have established a great efficiency among zebrafish in their capacity for cardiac regeneration throughout their life which can be harnessed to develop novel insights in investigating similar processes in humans [44,45]. Despite extensive injuries, zebrafish can regenerate a completely functional heart [46,47,48]. To date, zebrafish have demonstrated an extraordinarily robust response to cardiac damage compared to other vertebrates, as zebrafish can regenerate a complete heart even after the amputation of up to approximately 20% of the ventricle [11]. Although profuse bleeding occurs upon cardiac resection, a fibrin clot is quickly generated which stops exsanguination of the injured zebrafish [10,11]. In subsequent weeks, new muscle is observed to replace the fibrin clot with nearly total ventricular recovery between 1 to 2 months post-injury [10,11,49].

**Figure 1:**
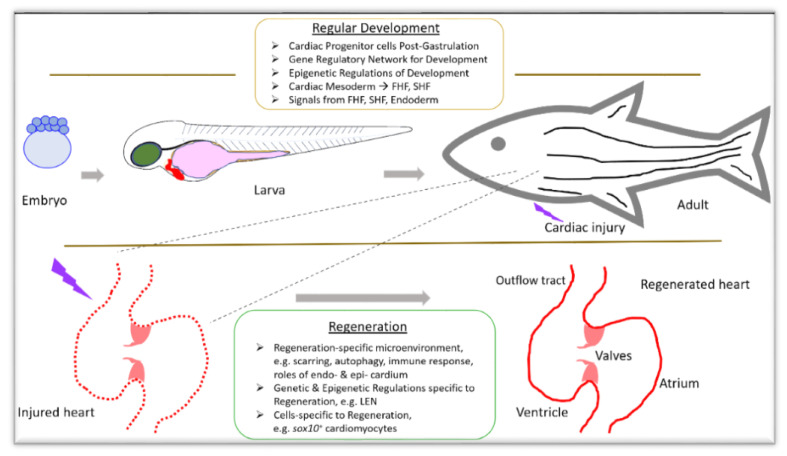
Overview of differences between cardiac development and regeneration in zebrafish. Upper and lower panels outline development and regeneration, respectively. Keys: FHF and SHF—1st and 2nd heart fields, LEN—*lepb*-linked enhancer, ➔—resulting in.

Interestingly, studies have reported that cells which contribute to cardiac repair and regeneration are different from cells that participate in processes of original cardiac development during embryogenesis [21] (Figure 1). Lineage tracing studies on cells have shown that cells from the myocardium, endocardium, etc., contribute to cardiac regeneration, but the process and underlying mechanisms are not clearly understood. However, studies have indicated that several genes regulate both cardiac development and regeneration through a tight orchestration of temporal and spatial regulation. In this direction, high-throughput studies investigating transcriptomic and epigenomic profiles during cardiac development and regeneration have the potential to provide novel insights into these processes. The discoveries will in turn improve our understanding of related processes in humans for therapeutic developments against congenital heart defects and cardiac injuries.

## 3. Zebrafish Supports State-of-the-Art High-Throughput Cardiac Research

### 3.1. Epigenomic Mapping of Zebrafish Reveals New Insights into Cardiac Development

Zebrafish epigenomic profiling was performed by Yuan et al., leading to the discovery of 5598 open chromatin regions that are conserved between zebrafish and humans, which indicates a diverse collection of ancient enhancers being established before organ development [50]. Genome-wide chromatin regions which are accessible or open to transcription were mapped using Assay for Transposase-Accessible Chromatin using sequencing (ATAC-seq) [51,52]. The study enabled Yuan et al. to identify enhancers in cardiac progenitor cells which become activated or expressed earlier than *Nkx2.5*, the cardiac progenitor marker whose mutations cause congenital heart disease in humans [53,54,55].

In this direction, Yuan et al. [50] engineered a green fluorescent protein (GFP) reporter line of zebrafish which is driven by murine Smarcd3 enhancer (Smarcd3-F6), because its activity is detected in early gastrulating mouse mesoderm [56,57,58]. Smarcd3 is also known as Baf60c in humans, a component of the Notch signaling pathway, which regulates cardiac looping morphology or left-right asymmetry [59], remodels chromatin to regulate gene expression for heart development and function [60,61], and determines the fate of cardiomyocyte cells [62]. Moreover, mutations in human *NOTCH1* result in aortic valve anomalies, such as severe calcification and stenosis, bicuspid aortic valve, ascending aortic aneurysm, atresia of the mitral valve, hypoplastic left ventricle, double-outlet right ventricle [63], and left ventricular noncompaction [64]. Mutations in the Notch ligand in humans, *JAG1*, are associated with Alagille syndrome, which contributes to several heart disorders [65,66].

In the study by Yuan et al., GPF-positive and GFP-negative cells were obtained from 10 hpf (hours post fertilization) zebrafish and subjected to ATAC-seq to detect enhancers that are active at a very early stage of development [50]. The results showed that the GFP-positive cells contain 155,879 ATAC-seq peaks which correlate to regions of open or accessible chromatin. The GFP-negative cells showed 153,777 peaks. Overall, significant quantitative differences were seen in 5471 peaks, among which 3838 were increased in GFP-positive cells and 1633 were increased in GFP-negative cells [50]. The ATAC-seq peaks showed significant overlap with histone modifications of H3K4me3 and H3K27ac, which mark active chromatin at promoters and enhancers, respectively. Prior ChIP-seq (chromatin immunoprecipitation) on zebrafish at similar developmental stages have validated H3K4me3 and H3K27ac as open chromatin marks [67].

Yuan et al. analyzed the ATAC-seq results using the Genomic Regions Enrichment of Annotations Tool (GREAT), which was previously developed to study the functional significance of cis-regulatory regions detected by localized quantitation of genome-wide DNA binding events [68]. GREAT showed that regions of open chromatin in the GFP-positive cells are enriched for processes related to heart development, including heart morphogenesis and embryonic heart tube formation. The distance of the regions of open chromatin from the genes of interest was also detected. In summary, the profiles of accessible chromatin that are enriched in GFP-positive and -negative 10 hpf zebrafish suggest that cardiac progenitor cells are marked by the Smarcd3-F6 enhancer. 

### 3.2. Transcriptomic and Chromatin Occupancy Profiling Zebrafish Reveals New Insights into Cardiac Regeneration

Enhancers are regions of accessible chromatin that are bound by transcription factors and histone modifications that activate transcription, e.g., histone H3 lysine (K) 27 acetylation (ac) [69,70]. Kang et al. investigated enhancer regulatory elements involved in tissue regeneration, using techniques including RNA-sequencing and ChIP-sequencing [71]. RNA-sequencing is a technique for quantification of all RNA transcripts and their sequence analysis [72]. ChIP-seq is a technique to map the genome-wide occupancy of proteins of interest and analysis of sequences where the proteins are localized [73]. 

Kang et al. performed transcriptomic analysis to screen for genes that are induced during regeneration in zebrafish. RNA was extracted from uninjured and injured fin and heart tissue of zebrafish, and subjected to library preparation, sequencing, and bioinformatics analysis. One of the genes that was found to be drastically upregulated upon transcriptomic analysis of regenerating caudal fins and cardiac tissue was the energy homeostasis regulator *leptin b* (*lepb*) [71]. Interestingly, the expression of leptin is upregulated in the failing human heart, suggesting a cardioprotective role [74]. Leptin has been further implicated in heart failure with preserved ejection fraction and left-ventricular hypertrophy [75,76], risk of coronary heart disease and stroke [77], and obesity-associated impairment of cardiac function and CVD [78,79].

Kang et al. [71] observed in zebrafish that partial resection-induced local injury of the cardiac ventricle resulted in *lepb* expression in the endocardium, which forms the endothelial lining of inner myofibers and participates in regeneration [80,81]. 

To detect enhancers that regulate cardiac regeneration and reside in the proximity of *lepb*, ChIP-seq for H3K27ac was performed in uninjured and injured regenerating zebrafish cardiac tissue. Chromatin from injured and uninjured ventricles was subjected to sonication, followed by immunoprecipitation with antibodies against H3K27ac, library preparation, sequencing, and bioinformatics analysis. Chromatin from the regenerating samples showed an enrichment of H3K27ac in two regions, 7 kb and 3 kb, upstream of *lepb* start codon, which was absent in the uninjured samples.

Further analysis revealed the region at 7 kb to be an enhancer because it showed strong blastemal expression upon fin amputation and strong fluorescence expression in the endocardium upon cardiac injury. Hence, ChIP-seq identified a short loci 7 kb upstream of *lepb* start codon as an enhancer of gene expression in adult tissues during regeneration. Further analysis of the ChIP-seq results revealed a short intergenic element called *lepb-linked enhancer* (LEN) which regulates regeneration-activated transcription from multiple promoters. Enrichment of H3K27ac during regeneration was also detected at several other 1–2 kb intergenic regions. Fins and hearts of transgenic zebrafish with *cmlc2* (marker of differentiated cardiomyocytes) or *α-cry* (lens marker) promoters also displayed robust fluorescence expression during regeneration, which indicates that these promoters have a role in the regeneration process. *cmlc2* is also known as *mlc7* or *myl7*, and it displayed endogenous post-transcriptional down-regulation in murine models of Down’s syndrome, which implicates this gene in human congenital heart defects that are detected in a majority of Down’s syndrome patients [82,83]. *MYL7* is also investigated in studies on cardiac biology using human pluripotent stem cells [84,85], cardiac regeneration in zebrafish [86], and cardiac failure [87].

### 3.3. CRISPR-Mediated Disruption of an Epigenetic Factor and Transcriptomic Analysis Reveal How a Signaling Pathway for Cardiac Development Is Impacted

RNA-sequencing of zebrafish has been extended to understanding the role of epigenetic factors in cardiac development. Epigenetic factors such as *KMT2D* and *KDM6A* are mutated in humans with Kabuki Syndrome, where a varying percentage of patients show cardiac defects [88]. Cardiac defects seen in Kabuki Syndrome patients include left-sided obstructions/aortic dilation, septation defects, coarctation of the aorta, etc. [89]. Studies have shown that disruptions of the zebrafish orthologs, *kmt2d* and *kdm6a*, recapitulate phenotypes of Kabuki Syndrome [88,90]. In this direction, Serrano et al. disrupted *kmt2d* in zebrafish by the elegant gene editing technology called CRISPR [91]. Next, they performed RNA-seq on single 1-day post-fertilization (dpf) zebrafish embryos with homozygous mutations in *kmt2d* and corresponding wild-types [90]. RNA was extracted, libraries were prepared and sequenced, followed by bioinformatics analysis. The timeline of 1 dpf was chosen so that alterations in pathways resulting in Kabuki Syndrome in *kmt2d* mutants can be detected immediately before the earliest phenotypes appear, because transcriptomic information at 1 dpf is not yet confounded by any of its own downstream effects.

The results showed differential expression of 276 genes on the mutants. Further analysis revealed that 72.1% of the top 50 genes are implicated in neural and/or cardiovascular systems, while the remainder are linked to reproductive system, muscle, and pharyngeal arches development [90]. For example, three genes detected in the analysis, *krt18*, *bgnb*, and *rbp7b*, are associated with human cardiac disorders. A study on humans and mice indicated *Krt18* as a cardioprotective factor induced by stress in failing hearts [92]. It is regulated by NHLRC2, whose mutations in humans cause severe fibrosis in tissues including the heart [93,94]. Fibrosis leads to loss and death of cardiomyocytes, leading to heart failure [95]. Loss-of-function mutations in *BGN* show a severe syndromic form of thoracic aortic aneurysms and dissections in humans [96]. *Rbp7* is expressed in the heart endothelium and other tissues [97], and likely regulates the antioxidant properties of the endothelium in association with PPARγ [98].

A small subset of genes appeared to be enriched during gene set enrichment analysis (GSEA), where one of the associations of each set was found to be structural extracellular matrix (ECM) glycoproteins. The observations indicate crucial variations in ECM content or topography prior to the onset of Kabuki Syndrome phenotypes. Interestingly, ECM is implicated in cardiac development, function, failure, and diseases [99,100,101,102].

The *kmt2d* mutants also showed an upregulation in *her4*.*4*, a specific downstream target of Notch, a signaling pathway conserved in metazoans that plays crucial roles in development including that of the zebrafish heart [49,90,103,104,105,106]. The observation is consistent with their finding of hyperactivation of Notch signaling in *kmt2d* mutants in the same study [90]. The transcriptomic profiles observed in 1 dpf zebrafish with *kmt2d* mutation support the mutant phenotypes observed at 3 dpf in this study. Such high-throughput transcriptomic analysis provides an excellent basis for discoveries on development and disease using zebrafish.

It is interesting to note that in addition to RNA-seq, the above study also employed CRISPR, adding to the growing popularity of zebrafish as a subject for CRISPR-mediated gene editing. Another study used CRISPR in a pioneering approach towards precise gene editing in zebrafish, to reveal cardiac developmental roles of a DNA-binding factor called *pbx3* [107], whose single nucleotide variant is associated with human disease. A cohort of patients with a mutation in *PBX3* showed a range of phenotypes for congenital heart defects including outflow tract malformations such as tetralogy of Fallot, atrioventricular septal defect, persistent truncus arteriosus, bicuspid aortic valve, coarctation of the aorta, and hypoplastic left heart syndrome [108]. The zebrafish study on *pbx3* identified its potential new role as a modifier in congenital heart defects [107]. Severe cardiac phenotypes were also observed upon CRISPR-mediated mutation of its family member, *pbx4*, which is known to regulate myocardial differentiation [109]. Patients with a mutation in *PBX4* showed an atrioventricular septal defect [108]. CRISPR-mediated gene editing of zebrafish extends to developmental studies beyond the heart, e.g., eye [110], craniofacial [111,112], muscle [113], etc.

### 3.4. CRISPR-Mediated Disruption of a Chromatin Modifier and Transcriptomic Analysis Reveal New Insights into Multiple Signaling and Biological Pathways Regulating Cardiac Development

Xiao et al. performed CRISPR-mediated disruption of another histone methyltransferase called *smyd4* in zebrafish, which disrupted histone modifications and caused severe cardiac defects [114]. *smyd4* is one of the five members of the Smyd family of genes which are lysine methyltransferases. Significant homology is maintained among all family members of Smyd proteins, from fish to humans [115]. Studies have implicated various members of the Smyd family in heart development and disease in different species. One member, *Smyd1*, shows a high upregulation in endogenous expression during human heart failure [115,116,117,118], which underscores the significance of the study by Xiao et al.

However, the roles of Smyd family members in adult heart development are not well-explored. In terms of genetic conservation for *smyd1*, it is known that zebrafish have *smyd1a* and *smyd1b* genes which could have resulted from whole-genome duplication in teleosts, mice have *Smyd1a*, *Smyd1b*, and *Smyd1c*, while humans have a single transcript from the *SMYD1* gene [115,119]. Other family members such as *smyd3* show modulation of mesodermal commitment during development in zebrafish and mice [120], while *smyd5* regulates hematopoiesis during zebrafish embryogenesis [121].

Xiao et al. used a CRISPR mutant of *smyd4* in a transgenic zebrafish line where cardiomyocyte marker *cmlc2* is labeled with green fluorescence protein. Transcriptomes of zebrafish hearts were analyzed to study how *smyd4* affects cardiac development. Zebrafish hearts were excised at 72 hpf based on the expression of green fluorescence, and RNA-seq was performed.

Differential expression of 3856 genes was observed in the mutant hearts, with upregulation in 2648 genes and downregulation in 1208 genes. Upregulation was seen in 10 genes associated with cardiac muscle contraction, as well as 36 and 15 genes associated with important cardiac signaling pathways such as canonical Wnt and Hedgehog, respectively. Downregulation was seen in 22, 10, and 3 genes which are respectively associated with cardiac muscle contraction, Wnt, and Hedgehog signaling pathways [114]. It is important to note that cardiac development and disease are associated with both Hedgehog [122,123,124] and Wnt signaling [125,126,127], with the reactivation of fetal non-canonical WNT gene program in human right ventricular failure [128].

Further analysis was performed to detect if the alterations in the transcriptome were linked to specific biological pathways. Analysis of KEGG (Kyoto Encyclopedia of Genes and Genomes) pathways indicated an enrichment of upregulated genes in the protein processing pathway of the endoplasmic reticulum in the Biological Processes domain [114]. Enrichment of downregulated genes was observed in the carbon metabolism and glycolysis/gluconeogenesis pathways. Enrichment of 975 genes in cellular metabolic processes was detected upon gene ontology analysis of upregulated genes.

Analysis of downregulated genes revealed enrichment of 185 genes in organonitrogen compound metabolic processes, 49 genes in ATP metabolic processes, and 55 genes in glycosyl compound metabolic processes. Overall, the results indicate that *smyd4* regulates epigenetic processes, which, as detected by the above analysis, likely results from an orchestration of the pathways involved in zebrafish cardiac development [114]. An interesting observation highlighted in this study is that the transcriptomic analysis of wild-type versus *smyd4*-mutant hearts showed differential expression of more than 3000 genes, but less than 100 genes among them have known roles in cardiac muscle contraction and cardiac signaling pathways, such as canonical Wnt and Hedgehog signaling. Instead, the analysis showed that several pathways of cellular metabolism were overwhelmingly enriched [114]. The observation steers our attention towards investigating links between metabolism and cardiac development in zebrafish, which are currently not well understood. The study further mentions that *smyd4* likely regulated cellular metabolism as one of its unique and specific biological functions [114]. Recent studies on the zebrafish heart indicate that metabolism regulates physiological phenomenon, including cell proliferation and differentiation, which crucially impact development and regeneration [129,130,131,132,133], and that antioxidants can be potential therapies against cardiac arrhythmia [134].

### 3.5. Lineage Tracing and Transcriptomic Analyses of Transgenic Reporter Zebrafish Reveals the Roles of a Distinct Subpopulation of Cells in Cardiac Regeneration

As mentioned earlier, the zebrafish model is also advantageous for research on cardiac regeneration using high-throughput methods involving next-generation sequencing. A study on cardiac regeneration by Sande-Melón et al. employed genetic fate mapping, transgenic reporter zebrafish lines, and RNA-seq to discover the role of a subset of cardiomyocytes in heart regeneration [135]. Sande-Melón et al. analyzed transgenic zebrafish and revealed that cardiomyocytes derived from *sox10* have distinct transcriptomic profiles compared to other cardiomyocytes. *sox10* is a transcription factor that crucially regulates the maturation of neural crest cells (NCCs) which are a population of precursors with stem cell-like properties originating in vertebrate embryos [136,137,138]. In humans, *SOX10* mutation impacts autonomic control of the heart dynamics [139], which indicates its role in heart innervation [140].

The study used hearts from adult transgenic zebrafish *sox10:CreER^T2^;vmhcl:loxP-tagBFP-loxP-mCherry-NTR*, which were subjected to cryoinjury or no injury. The rationale behind this transgenic line is explained below. Cre (causes recombination) is an enzyme that is classified as a recombinase or Type I topoisomerase and is a product of the bacteriophage P1 gene [141]. Cre causes site-specific recombination between loxP (locus of crossing (x) over, P1) sites in the genome [141]. Tamoxifen induces Cre/loxP recombination at embryonic stages in the *sox10:ER^T2^-Cre* zebrafish line as previously reported [142]. Hence, it is an efficient transgenic model to label NCCs using tamoxifen-induced Cre/loxP recombination, which helps to identify many established derivatives of NCCs in juvenile and adult zebrafish [142]. The recombination is induced upon treatment with an active metabolite of tamoxifen (4-OHT) that binds to estrogen receptors.

Other components of the transgene, such as *vmhcl* stands for ventricular myosin heavy chain-like; while *NTR* denotes nitroreductase; and *vmhcl:loxP-tagBFP-loxP-mCherry-NTR* (*vmBRN*) is known for specific tracing of *vmhcl*-expressing ventricular cardiomyocytes that emit red fluorescence after recombination [143]. It is interesting to note that zebrafish *vmhcl* is also known as *myh7l*, whose human ortholog is unknown, but human *MHY7* is associated with the heart. Mutations in *MYH7* are implicated in atrial fibrillation, which is common among hypertrophic cardiomyopathy patients [144], and *MYH7B* encodes a long non-coding RNA that significantly regulates the biology of cardiomyocytes [145].

In the transgenic zebrafish line used by Sande-Melón et al., the recombination induced by 4-OHT causes *sox10*-derived cardiomyocytes to express mCherry, or red fluorescence, while other cardiomyocytes express blue fluorescence. 4-OHT treatment for recombination was performed at different time points before cryoinjury, followed by harvesting the cardiac tissue at 7 days post-injury for transcriptomic comparison between injured hearts and uninjured hearts used as a control. Ventricles were obtained from uninjured and injured hearts which were subdivided into several pools followed by fluorescence-activated cell (FAC) sorting of 20 cardiomyocytes from each pool. RNA libraries were prepared, sequenced, and bioinformatics analysis was performed.

Cardiomyocytes derived from *sox10* or expressing red fluorescence in the uninjured ventricles showed upregulation of 101 genes and downregulation of 129 genes compared to the remaining cardiomyocytes [135]. Uninjured hearts showed metabolic contrasts between transcriptional profiles of *sox10*-derived cardiomyocytes and the remaining population upon gene enrichment analysis. Pathways diagnosed with differences include oxidative phosphorylation and nucleic acid metabolism [135].

In the injured hearts, *sox10*-derived cardiomyocytes showed higher transcriptional activity, with an upregulation of 415 genes compared to 30 genes in other cardiomyocytes. The injured hearts also showed a significant upregulation of *sox10* mRNA itself in *sox10*-derived cardiomyocytes. The observation verifies that cells expressing *sox10* can be endogenously traced using the *sox10:ER^T2^* line.

*sox10*-derived cardiomyocytes showed an upregulation of T-box transcription factors, *tbx20* and *tbx5a*, which are expressed in cardiomyocytes participating in cardiac regeneration [143] and actively regulate the regeneration of injured cardiac tissue [146,147]. In humans, *TBX20* mutations are associated with a spectrum of congenital heart defects including abnormalities in valvulogenesis, chamber septation, and growth [148]. Mutations in *TBX5* cause Holt–Oram syndrome, which includes cardiac defects in the conduction system, atrial and ventricular septation, and tetralogy of Fallot [149].

*sox10*-derived cardiomyocytes of injured hearts display a profile that favors regeneration when subjected to gene enrichment analysis, since they show inhibition of Gene Ontology (GO) biological processes associated with negative regulation of the cell cycle [135]. On the other hand, the cells show enrichment in pathways associated with the development of myocardium and cardiac cells, including cardiomyocyte differentiation and cardiac muscle contraction. The distinct transcriptomic profile of *sox10*-derived cardiomyocytes reported in the study confirms their crucial roles in regenerating the injured myocardium [135].

## 4. Conclusions

**Figure 2:**
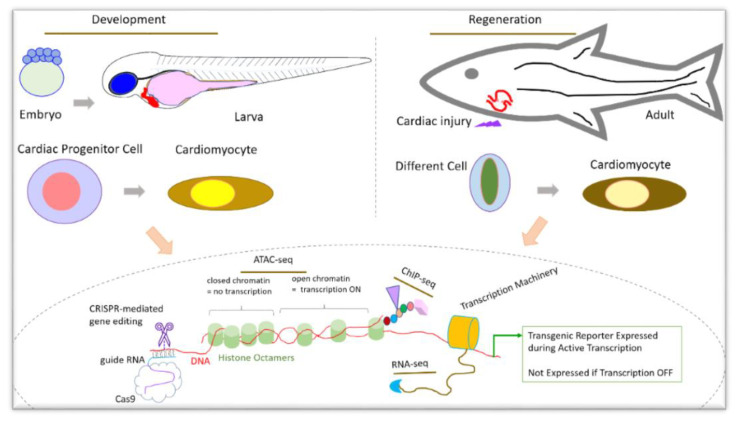
Zebrafish is an excellent model to study development and regeneration using state-of-the-art high-throughput technology. Zebrafish has several advantages to support ATAC-seq, RNA-seq, ChIP-seq, transgenic reporter line generation, gene editing by CRISPR, etc. Keys: ●●●—amino acid residues on N-terminal histone tails. □, Δ—histone modifications.

It is readily apparent that high-throughput techniques for genetic profiling are crucial to understanding heart development and regeneration (Figure 2). Considering the numerous orthologous genes related to cardiac function between zebrafish and humans, further applying these techniques to zebrafish as a model of study will undoubtedly lead to the discovery of the equivalent processes in humans. Two zebrafish-based clinical trials against heart disease are already underway, (NCT04009759) and (NCT02641145), where the latter has collaborations with the American Heart Association and the National Institutes of Health (NIH). Although significant discoveries have been made in the field of cardiac development and regeneration, our understanding of the mechanisms behind their similarities and differences is not completely clear.

In addition to developmental gene regulatory networks, considerable impact of epigenetics [17], distinct cell types, and signaling pathways require more thorough investigations using high-throughput technologies. For a better understanding of regeneration responses so that human cardiac injuries can be addressed, further focus is needed on the regeneration microenvironment [19,22] and cellular roles [150,151,152,153]. These discoveries may, in turn, illuminate new pathways for the early diagnosis of damage to cardiac tissue as occurs in heart disease, such as through the identification of relevant promoters [50], enhancers [50,68], and transcription factors [114,135], as well as new methods of treatment to encourage regeneration of cardiac tissue. To address the above questions, advanced high-throughput genome-wide profiling of transcriptome, epigenome, proteome, metabolome, etc. would be extremely beneficial.

As evidenced by the studies discussed in this review, zebrafish serve as an excellent model system for the study of cardiac development, and regeneration due to its multifaceted ability to regenerate cardiac tissue, as well as the species’ compatibility with efficient and accurate high-throughput research techniques.
